# A phylogenetic comparative analysis on the evolution of sequential hermaphroditism in seabreams (Teleostei: Sparidae)

**DOI:** 10.1038/s41598-020-60376-w

**Published:** 2020-02-27

**Authors:** Susanna Pla, Chiara Benvenuto, Isabella Capellini, Francesc Piferrer

**Affiliations:** 1 Institut de Ciències del Mar, Spanish National Research Council (CSIC), Barcelona, Spain; 2 Ecosystems and Environment Research Centre, School of Science, Engineering and Environment, University of Salford, Salford, UK; 3 School of Biological Sciences, Queen’s University of Belfast, 19 Chlorine Gardens, Belfast, BT9 5DL UK

**Keywords:** Evolutionary theory, Ichthyology

## Abstract

The Sparids are an ideal group of fishes in which to study the evolution of sexual systems since they exhibit a great sexual diversity, from gonochorism (separate sexes) to protandrous (male-first) and protogynous (female-first) sequential hermaphroditism (sex change). According to the size-advantage model (SAM), selection should favour sex change when the second sex achieves greater reproductive success at a larger body size than the first sex. Using phylogenetic comparative methods and a sample of 68 sparid species, we show that protogyny and protandry evolve from gonochorism but evolutionary transitions between these two forms of sequential hermaphroditism are unlikely to happen. Using male gonadosomatic index (GSI) as a measure of investment in gametes and proxy for sperm competition, we find that, while gonochoristic and protogynous species support the predictions of SAM, protandrous species do not, as they exhibit higher GSI values than expected even after considering mating systems and spawning modes. We suggest that small males of protandrous species have to invest disproportionally more in sperm production than predicted not only when spawning in aggregations, with high levels of sperm competition, but also when spawning in pairs due to the need to fertilize highly fecund females, much larger than themselves. We propose that this compensatory mechanism, together with Bateman’s principles in sequential hermaphrodites, should be formally incorporated in the SAM.

## Introduction

Among vertebrates, fishes exhibit the broadest diversity in sexual systems^1–3^, ranging from gonochorism (separate sexes) to hermaphroditism (sequential and simultaneous), unisexuality (all individuals are females) and mixed sexual systems (i.e., co-occurrence of hermaphrodites and males, as in *Kryptolebias* killifish^4,5^). Sex allocation theory aims to answer questions such as what allocation of resources to males and females is favoured in gonochoristic species, when and in which direction to change sex in sequential hermaphrodites (from male to female in protandry and from female to male in protogyny), how much energy should be devoted to male *vs*. female function in simultaneous hermaphrodites, and under what ecological conditions these different sexual systems are evolutionary stable strategies^6^. Thus, understanding the evolutionary drivers and consequences of the transitions from one type of sexual system to another has been of great interest since the early days of the study of the evolution of sex^7,8^.

Gonochorism is believed to be the ancestral condition from which all other sexual systems evolved in fish^9–11^. Recent phylogenetic reconstructions of the evolutionary history of sexual systems in some species-rich families, though, have found ancestral protogyny in Labridae^12^ (where species are gonochoristic, protogynous and bi-directional sex changers) and in Serranidae^13,14^ (where species are gonochoristic, protogynous and simultaneous hermaphrodites)^14^. However, an earlier study using parsimony could not resolve the ancestral state for sexual system in the family Sparidae (porgies or seabreams). The Sparidae are a diverse, monophyletic group^15,16^ consisting of ~150 coastal fish species^17^ with a wide geographical distribution, mainly in tropical and temperate waters^18^. This group is an ideal taxon to study the evolutionary history and adaptive significance of sexual systems, particularly for both types of sequential hermaphroditism, given that it contains many gonochoristic, protogynous and protandrous species, with differences in sexual systems even between species belonging to the same genus.

The main theoretical framework to explain the evolutionary advantage of sequential hermaphroditism based on sex allocation theory is the size-advantage model (SAM)^9,19–21^. The SAM proposes that individuals should switch sex when the second sex achieves a greater fitness at a larger body size than the first sex, thus increasing lifetime fitness^22^. When larger males have higher reproductive success than smaller males and similar sized females, fish should reproduce initially as female and change later to male (protogyny)^19,23,24^. Conversely, protandry is expected when large females have greater fitness than smaller females and similar sized males^19,20^. Empirical studies support this model in several fish families, explaining quantitatively the size or age at which an individual should change sex, as well as the overall population sex ratio^25,26^. Typically, male-biased sex ratios are observed in populations of protandrous species, while female-biased sex ratios are observed in populations of protogynous species, especially in haremic groups^19,26,27^.

Mating systems (the number and social dominance of mates involved) and spawning mode (i.e., group spawning, where several males spawn with one or several females, or pair-mating, where a single male spawns with a single female), determine the fitness that a fish of a given size can achieve as a female or as a male^23,28^. Fish mating systems range from monogamy or pair-mating, to harem polygyny (one dominant male controls access to multiple females^29^), lek-type polygyny (temporary harems^25^), and large polygamous or promiscuous aggregations (where group spawning occurs^30^). Sperm competition is particularly intense in large aggregations with multiple males^31,32^ while it is absent in monogamy and pair-mating, and low in polygynous mating systems. Sperm competition and spawning modes have been used to infer mating systems^33–35^, which are relevant when testing the predictions of SAM since they can generate sex-specific differences in reproductive expectations^23,24,36,37^. Thus, the first study that tested the predictions of SAM incorporating the mating system in a phylogenetic context focused on the evolution of protogynous groupers according to pair spawning mode^35^. Indeed, protandry should be favored when sperm competition is low (mating occurs between members of monogamous or random pairs^19,22–24^), and mate monopolization by large males should occur in protogynous species^23^. Even though sperm competition was not included in the original formulation of the model^9,19^, it was subsequently mentioned as absent in protogyny^21,23,24,38^, limited in protandry (considered typical of random pairing and small groups)^22,23^ and high in gonochorism (when mating occurs in large groups)^21,23,35,39^. The influence of sperm competition on sex change has been tested, under the SAM framework, in 116 protogynous and gonochoristic species^40^ confirming smaller relative testis size in protogynous sex changers compared to gonochoristic species^19,38,40,41^.

The combined effect of sexual and mating systems on reproductive success has been reported not only in many fish families, including Serranidae, Labridae, Platycephalidae, Pomacanthidae, Pomacentridae and Gobiidae^13,14,19,23,35,37,42,43^, but also in other taxa, e.g., crustaceans^44,45^ and molluscs^46–48^. Overall, the SAM is well supported^12,37^, but in some cases model expectations do not hold when tested in the field^22,49^. Nevertheless, the SAM provides an elegant and simple evolutionary framework to understand the adaptive significance of sex change, particularly when integrating relevant factors that could be implicated in the evolution of sexual systems^19,35^.

In this study, we follow the idea of previous studies to consider mating systems^14,35,40^ as a starting theoretical framework of SAM by including explicit predictions for spawning mode, mating system and sperm competition for both protogynous and protandrous systems (Table 1). Using this broader model’s perspective, protogyny should be favoured when males can dominate a social group, with stable or temporary territorial males (e.g., in lek) that monopolize mating harems^22,23^. These social systems diminish sperm competition due to the absence of group spawning (as found in many members of the family Serranidae^23,24,38,50–52^). The intensity of sperm competition that a male fish faces can be reliably estimated by the gonadosomatic index (GSI), a proxy of gamete production^38,53^. The GSI is defined as the proportion of gonad weight relative to total body weight^32^. Thus, high GSI values (>3%) in males are typically observed in species that spawn in large aggregations^54,55^, where males compete at the post-copulatory stage for fertilizing the eggs, as it occurs in many pelagic gonochoristic spawners. Thus, SAM proposes that protogyny is adaptive when there is low sperm competition and as a consequence or and thus low GSI values^51,52^. Because gonochoristic species can have either high or low sperm competition levels, as they exhibit a variety of mating systems and spawning modes, their GSI should be on average higher than that of protogynous species^19,35,38,40,41^ (Table 1).

**Table 1:** Simplified characterization of mating systems (monogamous, polygynous, promiscuous; see text for full definitions), spawning mode (pair spawning or group spawning), sperm competition (classified as low or high) and gonadosomatic index, GSI (classified as low or high) for each sexual system (G = Gonochorism; PA = Protandry; PG = Protogyny). In bold: most common type.

Sexual System	Mating System	Spawning mode	Sperm competition	GSI	Prediction
PG	**Harem polygyny**	**Pair**	**Low**	**Low**	Low
	**Lek polygyny**	**Pair**	**Low**	**Low**	
	Promiscuous large groups*	Group	High	High	
PA	**Monogamy**	**Pair**	**Low**	**Low**	Low
	**Random pair mating**	**Pair**	**Low**	**Low**	
	**Promiscuous small groups**	**Small group**	**Low**	**Low**	
	Promiscuous large groups**	Group	High	High	
G	**Promiscuous large groups**	**Group**	**High**	**High**	High
	Monogamy (size assortative mating)	Pair	Low	Low	
	Monogamy (random pair mating)	Pair	Low	Low	

The prediction column summarizes the overall expectation on sperm competition (and thus GSI) for the sexual system, based on the most common mating and spawning mode, according to the SAM^23,24^.

*Protogynous group spawners should be favoured as large males can produce more sperm^28^.

**Promiscuous large groups are not common, but still present in protandrous species (see text for examples).

While direct male dominance should select for protogyny, the combined effects of the increase of female fitness with size and the interaction of just few males resulting in low sperm competition (also known as ‘budget effects’) should select for protandry^56^ (Table 1). Although we know little about mating systems in protandrous species, at least a few sparids appear to conform to these predictions (e.g., *Sparus aurata*^57^; *Rhabdosargus sarba*^58^) as they have been mainly reported to mate in pairs or small groups, thus in the absence of multiple males competing among themselves^57,58^. Therefore, we can expect low levels of sperm competition in protandrous sparids^22–24^ and consequently low GSI values. However, a handful of protandrous species engage in random matings in large spawning aggregations (e.g., *Acanthopagrus berda*^59^)^19^ where sperm competition should be intense, and thus higher GSI values than pair-mating species should be expected. Due to this diversity of mating systems, a wide range of GSI values has accordingly been observed in protandrous Sparidae^14^. Altogether, the SAM incorporating sperm competition and mating systems appears to be well supported in the Sparidae, with protogynous species exhibiting low levels of sperm competition as predicted^14,35,40,54^; however, protandrous species exhibit much higher GSI values on average than SAM predictions suggesting that they face more intense sperm competition than previously thought^14^. As Warner^24^ stated (page 88–89), we “will have to go beyond the mating system to discover the actual size-specific reproductive expectations in protandrous species”.

A previous study^14^ based on recorded GSI values suggested high level of sperm competition in some protandric sparids but did not formally test nor discuss the potential confounding effects of mating system, spawning mode and allometry. Although GSI, the percentage of gonad tissues on body mass, may provide an estimate of investment in the gonads irrespective of size, it is possible that at least some of the observed differences in male GSI across species are in part determined by differences in male size, if investment in the gonads is easier at larger or smaller sizes. In addition, the reported high GSI values in this study were derived from only eight protandrous species^14^, three of which were in fact those of individuals reproducing as female rather than male, or, unlike for other species in the dataset, do not correspond to the maximum recorded male GSI value for the species and so are not comparable. Finally, recent phylogenetic trees, which are essential for all comparative studies are better resolved and more comprehensive than those employed by earlier studies (e.g.^18^), while modern phylogenetic comparative methods allow overcoming the limitations of approaches used in previous studies, such as parsimony reconstruction and phylogenetic independent contrasts, that can lead to incorrect conclusions^60,61^.

Here we have compiled the largest dataset to date of sexual systems, spawning modes and body size in the family Sparidae. Using modern phylogenetic comparative approaches, we have investigated the evolutionary history of sexual systems and tested the predictions of the SAM, considering spawning mode and mating systems, that protogynous and protandrous species should exhibit lower GSI values due to expected lower sperm competition (Table 1) than gonochoristic species, while accounting for body size. Our study therefore combines sexual systems, mating systems, sperm competition and the principles of the SAM in several sparid species, while accounting for the potential confounding effects of allometry and phylogeny.

## Methods

### Data collection

We used FishBase (www.fishbase.org^62^) to gather information on the sexual systems of Sparidae, ranging from gonochorism (G), protandrous hermaphroditism (PA) and protogynous hermaphroditism (PG). We verified, and if necessary corrected, the sexual system reported in FishBase for each individual species used in this study against the primary literature^14,25,54,63,64^. We carefully revised previous assignments of sexual systems in four species, which were mainly based on the gonadal morphology of individuals collected at single or different ages^65^. Sometimes this approach cannot distinguish functional (active) hermaphrodites from non-functional hermaphrodites (i.e., individuals that despite presenting both male and female tissues reproduce only as one sex throughout their life). The assignment of the correct sex is further complicated in non-reproducing juveniles, which can present a bisexual gonadal stage. Altogether, these peculiarities can make diagnosis of sexual system extremely challenging^14,54,64^. We resolved any discrepancies from previous studies using newly published data in which care was taken not to incur in the above problems (Table S1).

Out of a total of 148 recognized sparid species, we could assign the sexual system in 68 species (Table S1). For these species, we extracted data from FishBase on male maximum body weight (in g; n = 37 spp.), male maximum total body length (in cm; n = 47 spp.) and male total body length at maturity (in cm; n = 36 spp.), to account for possible allometric effects on GSI. Finally, we extracted data from the primary literature on male GSI (n = 49) and spawning mode (pair or group spawning, based on the presence of one or more males, respectively; n = 10 spp.). When several GSI values were reported for a given species (e.g., monthly means along the year), we consistently used the highest value.

### Ancestral state reconstruction

We used two molecular phylogenetic trees with time-calibrated branch lengths, an essential step for robust analyses in a phylogenetic comparative framework^66^. Specifically, we used a phylogeny of Actinopterygians^67^ based on a 27-genes (6 mitochondrial and 21 nuclear genes) and a phylogeny for the family Sparidae^68^, based on three mitochondrial and two nuclear genes. These trees included 58 and 55 species, respectively, out of the 68 species with sexual system information in our dataset.

The ancestral state reconstruction infers the evolutionary history of a trait along a phylogeny given the character states of species in the tree and provides estimates of the probable character state of each node in the phylogeny. This approach is based on a Markov model of evolution for discrete traits^69^. We reconstructed the ancestral character states of sexual system using maximum likelihood (ML), setting all transition rates between G, PA and PG free to vary, i.e. they are not constrained to be of equal magnitude. We ran these analyses on both phylogenetic trees using the R package *ape*^70^. However, we can only report the results of the ancestral state reconstruction using the phylogenetic tree of Rabosky *et al*.^67^ since the analysis using the Santini *et al*.^68^ tree did not converge to a maximum likelihood solution.

### Testing SAM predictions

We used phylogenetic generalized least square models (PGLS^61,71,72^) to test the predictions of the SAM using the R package *caper*^73^ and ML estimation, with both phylogenetic trees^67,68^. By incorporating the phylogeny, PGLS models can quantify the strength of phylogenetic signal in the data through the parameter lambda (λ), which can vary between zero (no phylogenetic signal) and one (high phylogenetic signal, whereby the species exhibit phenotypic similarity in direct proportion to their common evolutionary time)^61,72^. GSI was entered as the dependent variable, while sexual system was the independent discrete variable with three possible states (0 = G, 1 = PA, 2 = PG), and body size (as maximum length, weight or length at maturity) the independent continuous covariate. Possible allometric effects on the GSI were thus accounted for using either body length or weight as additional independent variable. Continuous variables were log_10_-transformed to meet assumptions of normality, with the exception of GSI values. Results for GSI were qualitatively similar whether this variable was log_10_-transformed, transformed with logit function or left untransformed, thus we report the results of GSI in percentage, not transformed. All model residuals were normally distributed in all analyses.

We also tested the SAM prediction within the genus *Diplodus*. This was the only genus that provided limited but sufficient data to consider at least two different sexual systems (G *vs*. PA) for statistical analysis of the relationship between sexual system and male GSI values in very closely related species. Importantly, *Diplodus* species have a narrow range of body sizes and thus there is less variability and potential confounding effects of allometry. However, the analysis could not be carried out in a phylogenetic context in this genus because too many species were missing from both phylogenies. Therefore, we used Student’s *t*-test for independent samples to test differences in total male body length and GSI between the two sexual systems, and general linear model to test for the relationship between maximum male length at reproduction and GSI. In all analyses, performed in R^74^, differences were considered statistically significant when *p* < 0.05.

## Results

Our dataset on the sexual system of 68 sparid species across 28 genera (out of 37) includes 27 gonochoristic, 22 protogynous and 19 protandrous species (Table 2, Tables S1 and S2), with the three sexual systems roughly present in similar proportions (range~28–40%; Table 2). This represents a substantial increase in the number of species previously investigated^14^.

**Table 2:** Distribution of the major types of sexual systems among sparids, indicating absolute numbers and percentages with respect to all fishes in the family.

	Genera	Species
Total number	37	148
With known sexual system	28 (73.68%)^a^	68 (45.94%)^a^
Gonochorism	20 (68.96%)^b^	27 (39.70%)^b^
Protogyny	12 (41.37%)^b^	22 (32.35%)^b^
Protandry	8 (27.58%)^b^	19 (27.95%)^b^

^a^Percentage with respect to total number.

^b^Percentage with respect to number with known sexual system. Percentages add to 100 only for species with known sexual system since in some genera different sexual systems can be present.

**Figure 1:**
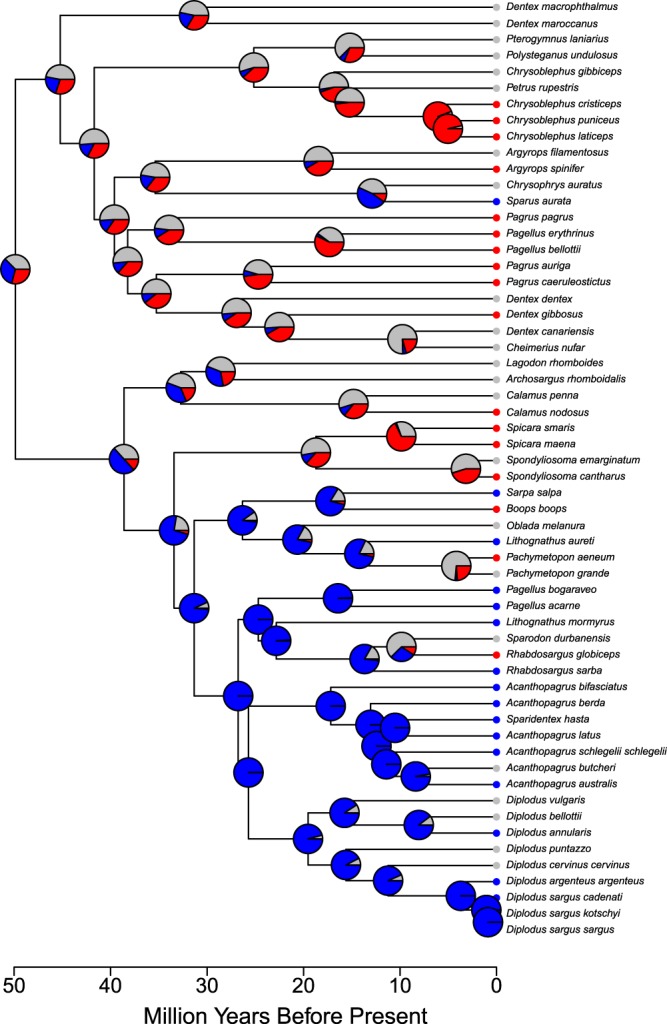
Ancestral state reconstruction in the Sparidae using the phylogenetic tree by Rabosky *et al*.^67^. Sexual system is coded as gonochorism (grey), protandry (blue) and protogyny (red). The pie area indicates the likelihood of character state at each node for the three states.

**Figure 2:**
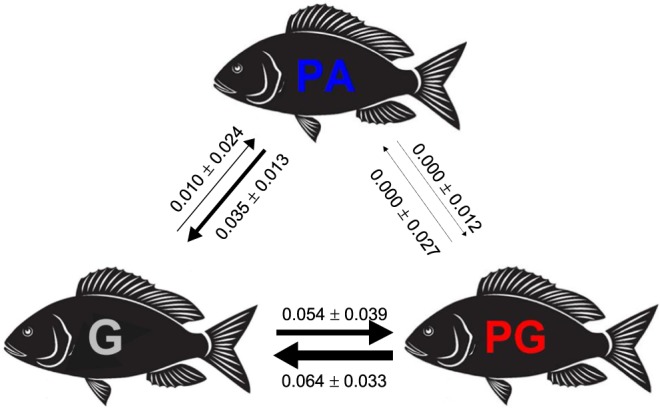
Transitions rates between sexual systems in the Sparidae derived from the ancestral state reconstruction in maximum likelihood. G: gonochorism; PA: protandry; PG: protogyny.

Reconstruction of the ancestral character state in a phylogenetic context showed that gonochorism was only slightly more likely to be the ancestral sexual system in the Sparidae family (likelihood at the root 37.4%) than protandry (33.5%) and protogyny (29.1%; Fig. 1). While both forms of sequential hermaphroditism, especially protogyny, evolved rapidly to gonochorism (PA to G: 0.035 ± 0.013; PG to G: 0.064 ± 0.033), gonochorism reverted as quickly back to protogyny (G to PG: 0.054 ± 0.039) and much less so to protandry (G to PA: 0.010 ± 0.024). Finally, the transitions between the two forms of sequential hermaphroditism were both estimated to be zero (PA to PG: 0.000 ± 0.012; PG to PA: 0.000 ± 0.027; Fig. 2).

**Figure 3:**
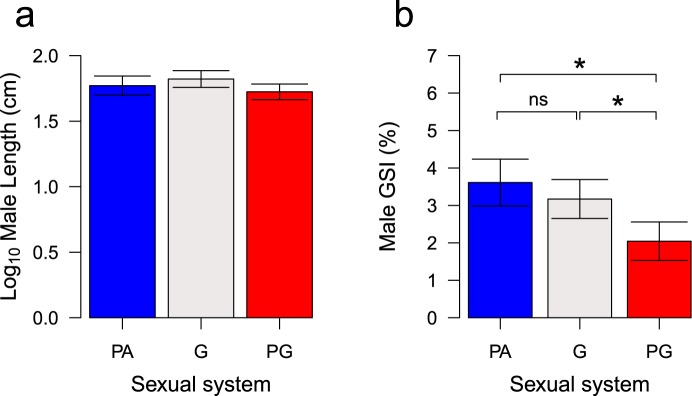
Phylogenetic means and standard errors of (**a**) Log_10_ Male total length (G: n = 19; PA: n = 12; PG: n = 10), (**b**) GSI (G: n = 15; PA: n = 15; PG: n = 14) across sexual systems (G: gonochorism; PA: protandry; PG: protogyny). Asterisks indicate statistically significant differences with the following equivalence: *p < 0.05.

**Figure 4:**
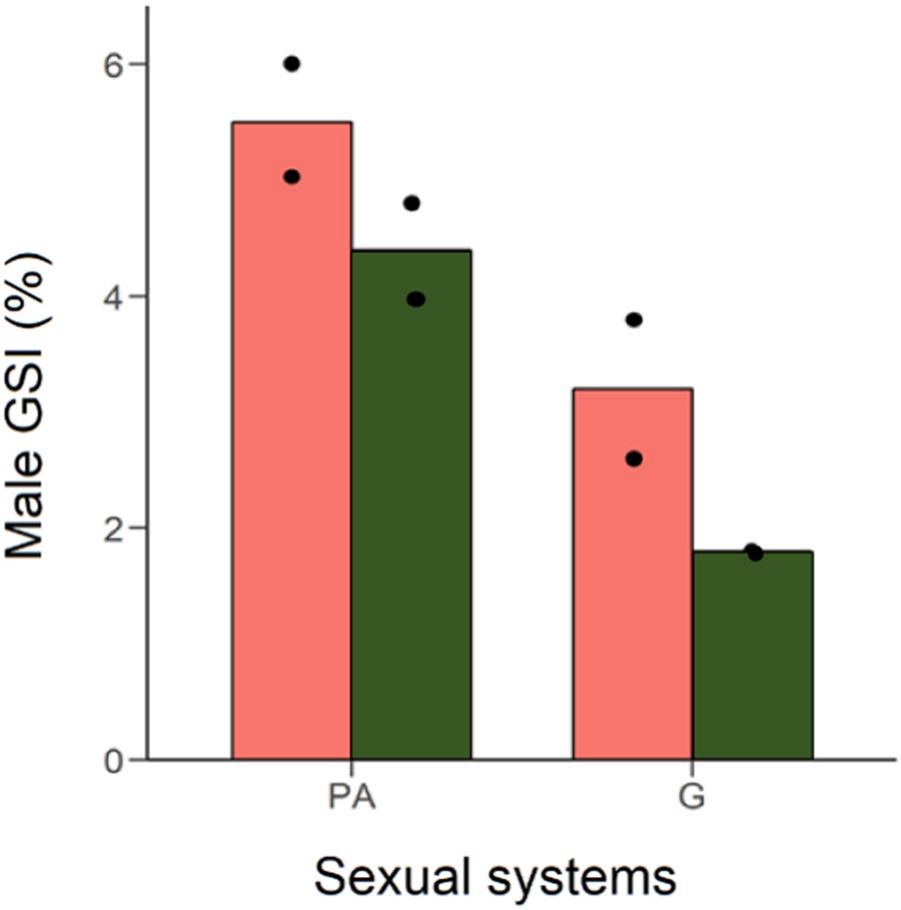
Mean of male gonadosomatic index (GSI) in protandrous (PA) *vs*. gonochoristic (G) species of Sparidae as a function of their spawning mode (Red, group spawning; Green, pair mating). The black dots indicate individual values in species with information for the three variables: sexual system, spawning mode and male GSI.

We did not find any significant difference in total male body length (Fig. 3a), male length at maturity or maximum male body weight between sparid species with different sexual systems (Table 3). For the 46 sparid species in the tree where male GSI values were available (Table S3), GSI values of protandrous and protogynous species were higher and lower respectively than that of gonochoristic species, with statistically significant differences between protogyny and the other two sexual systems (Fig. 3b; Table 3). Results were qualitatively similar regardless the phylogenetic tree used (Table S4) and were not influenced by allometric effects, tested using either length or weight as a covariate in the model (Table S5). Unfortunately, limited data were available on spawning mode, for both species that spawn in groups (G, n = 2; PA, n = 4; PG, n = 0) or in pairs (G: n = 1; PA: n = 2; PG: n = 1). These low numbers did not allow testing predictions for mating systems formally. However, the data appear to suggest that protandrous sparids have higher GSI values than gonochoristic species regardless of whether they spawn in groups or pairs (Fig. 4).

**Table 3:** Phylogenetic analysis of male life-history traits (see text for full definition) according to sexual system (G = Gonochorism; PA = Protandry; PG = Protogyny) using the Rabosky *et al*.’s^67^ phylogenetic tree.

Variables		Beta	T	P	df	Model stats	
Dependent	Independent					λ	R^2^
Weight (log_10_ transformed)	Sexual system – PA^a^	0.11	0.77	0.44	2,29	1.00	0.06
	Sexual system – PG^a^	−0.19	−1.07	0.29	2,29		
	Sexual system – PG^b^	−0.30	−1.41	0.16	2,29		
Length (log_10_ transformed)	Sexual system – PA^a^	−0.04	−0.77	0.44	2,44	0.71	0.06
	Sexual system – PG^a^	−0.09	−1.64	0.10	2,44		
	Sexual system – PG^b^	−0.04	−0.61	0.54	2,44		
Length Maturity (log_10_ transformed)	Sexual system – PA^a^	−0.06	−0.85	0.39	2,24	1.00	0.03
	Sexual system – PG^a^	−0.05	−0.65	0.51	2,24		
	Sexual system – PG^b^	0.01	0.13	0.89	2,24		
GSI (%)	Sexual system – PA^a^	0.43	0.84	0.40	2,43	0.70	0.14
	Sexual system – PG^a^	−1.12	−2.19	**0.03**	2,43		
	Sexual system – PG^b^	−1.56	−2.55	**0.01**	2,43		

Notes: Gonochorism in^a^ and protandry in^b^ were set as the reference level, respectively.

Abbreviations: T: t-value; df: degrees of freedom; P: *p*-value; λ: phylogenetic signal. Significant differences (*p*-values < 0.05) are in bold.

**Figure 5:**
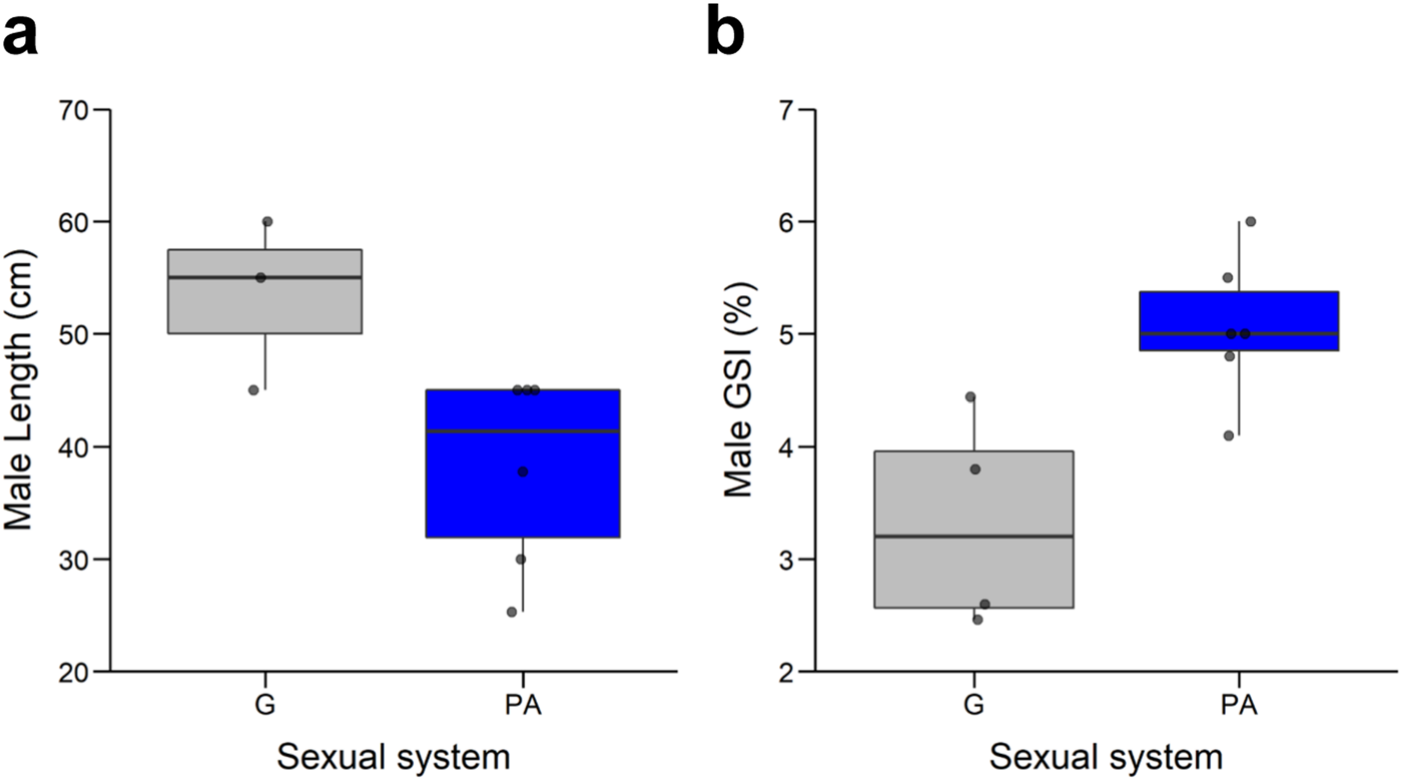
Maximum length and gonadosomatic index (GSI) of males in the genus *Diplodus* as a function of their sexual system (G = Gonochorism; PA = Protandry). (**a**) Maximum length (G: n = 3; PA: n = 6). (**b**) GSI (G: n = 4; PA: n = 6). The lower and upper edges of the boxes indicate the lower and upper quartiles, respectively; upper whisker = min (max(x), Q3 + 1.5 * IQR); lower whisker = max (min(x), Q1–1.5* IQR, where IQR = inter-quartile range, defined as the third quartile (Q3) – first quartile (Q1). The median is indicated by solid black horizontal line. The black dots indicate individual values. Asterisks indicate statistical significant differences: *p < 0.05.

We then repeated the analyses within the genus *Diplodus*, which contains only gonochoristic and protandrous species with a narrower range of lengths (~25–60 cm) when compared to that of sparids as a whole (~20–200 cm). We found no significant differences in weight (*t*_2*.98*_ = −0.54; *p* = 0.62) between gonochoristic and protandric species. However, the latter had a significantly shorter length than the former (*t*_*4.61*_ = 2.71; *p* = 0.04; Fig. 5a). Importantly, despite being smaller in size, protandric *Diplodus* species had a significantly higher GSI (*t*_*4.82*_ = −3.19; *p* = 0.02) than the gonochoristic species of the same genus (Fig. 5b), confirming the results found across all sparid species. Moreover, GSI was unrelated to male total body length (F = 11.65; df=2,6; *R*^*2*^ = 0.79; *p* = 0.246), suggesting that GSI differences between sexual systems were not determined by allometric effects.

## Discussion

With a larger dataset of sexual systems in the family Sparidae than previously used, this study reveals that protandry and protogyny can evolve from gonochorism, although the ancestral state remains still uncertain in this family. Importantly, we find that transitions between the two forms of sequential hermaphroditism are unlikely, if ever, to occur. We find strong support for the SAM predictions, incorporating mating system and sperm competition, that protogynous species should exhibit lower levels of sperm competition relative to gonochoristic species (Table 1), as quantified by their low GSI values, consistent with their mating systems that allow large males to monopolize access to fertile females. However, we find no evidence in support of similar predictions in protandrous sparids, i.e., low levels of sperm competition because they are expected to spawn in pairs or small groups (Table 1). Unexpectedly, protandrous species have similar GSI values to those of gonochoristic species and higher GSI than protogynous species, regardless of their mating system. Below we propose how a compensatory mechanism, together with mating system and spawning mode, may explain this unexpected finding.

Using twice as many species relative to an earlier study, recent molecular, time-calibrated phylogenies and modern phylogenetic comparative approaches, our study shows that gonochorism is only marginally more likely to be the ancestral state in this family. We find that gonochorism can evolve into both protogyny and protandry. However, sequential hermaphroditism is an evolutionary unstable state as it reverts quickly back to gonochorism, suggesting that both types of sequential hermaphroditism are costlier to sustain than gonochorism. These results may explain why, despite hermaphroditism being anatomically and physiologically possible in fish in contrast to other vertebrates^75^, gonochorism predominates among fishes^64^. Female has been often considered the ‘default sex’ in fishes because, even in some gonochoristic species, all individuals, exhibit ovarian differentiation at the early stages of development, a process that is subsequently halted in the individuals that become males^63,76^. Thus, it is perhaps not surprising that our analysis reveals that the evolutionary transition rate from gonochorism to protandry is very low and that transitions from protogyny to protandry and *vice versa* are unlikely to occur; once canalized towards initial development as a female, it may be too costly to switch the developmental pathway to male-first sex changer (and *vice versa*).

We tested whether the sparids conform to SAM predictions when incorporating sperm competition, mating system and spawning mode as previously done in protogynous and gonochoristic epinephelids^35^. Specifically, we tested whether gonochoristic species, which often spawn in large groups or aggregations and are typically characterized by intense sperm competition, have a higher GSI than species with either types of sequential hermaphroditism. Sperm competition is indeed less intense in haremic species (often protogynous), where the presence of few dominant large males drastically reduce the interaction between sperm of different males^24,35,40^. It is also generally accepted that protandrous hermaphrodites normally reproduce in small, random mating groups (no size-assortative) or in strictly monogamous pairs^23,24,77^ as, for example, in most clownfish (family Pomacentridae) of the genus *Amphiprion*, such as *A. melanopus* and *A. percula*^78–80^. In both mating systems, this would imply a low degree of sperm competition and thus low values of GSI, as predicted by the SAM^22–24^. As expected, we found that the average male GSI in gonochoristic sparids was >3%, in agreement with previous reports^14,35,40^, and significantly higher than the mean GSI of protogynous sparids (≤2%). These results, therefore, support the SAM prediction, incorporating sperm competition, in protogynous species (Table 1). However, our study also reveals that protandrous sparids have, on average, the highest male average GSI value (3.6%), regardless of their mating system. Thus, even when mating in pairs or small groups, protandrous males invest heavily in the gonads, indeed even more than gonochoristic species that mate in large aggregations with intense sperm competition. Further, we demonstrate that these results are not determined by differences in body size, as we find no allometric effects on GSI values, neither across all species, nor within one genus (*Diplodus*) containing closely related species of similar size with different mating systems. Altogether, our study unambiguously demonstrates that, while gonochoristic and protogynous sparids conform to a broader version of the SAM including sperm competition, spawning mode and mating system, protandrous sparids do not. We suggest that these results may be explained by both spawning mode and a compensatory mechanism determined by high sexual size dimorphism (SSD) in protandrous species. In protandrous species, sexual size dimorphism has been confirmed in several species such as *Diplodus annularis* and *Lithognathus mormyrus*^81,82^. However, when we checked male GSI in relation to SSD in the few protandrous fishes for which there is information we found a weak (r^2^ = 0.3) positive relationship that nevertheless was not significant probably due to low sample size (data not shown). Therefore, the high GSI in protandrous species is not, in principle, explained by the magnitude of SSD. However, more data to would be needed to confirm this observation.

Some protandrous sparid species like *Acanthopagrus berda, Sarpa salpa*^83^, *Diplodus capensis*^84^ and *D. annularis*^81^, spawn in aggregations^59^, similarly to many gonochoristic species^14^ and should therefore experience intense sperm competition, leading to a high GSI value. Indeed, we often tend to oversimplify the complexity of sequential hermaphroditism: not all protogynous species are haremic, as not all protandrous species mate in pairs. A recent study^28,85^ has revealed a broader variation in effective population size in protogynous species that differs from the more limited expectations obtained when all protogynous species were considered haremic by default. Instead, some protogynous species were found to be group spawners, altering the expectations that all protogynous species should have low effective population size due to their supposed haremic system. Similarly, not all protandrous species are monogamous or mate in pairs and small groups. Furthermore, many of the protandrous sparids that engage mostly or exclusively in pair mating, including *Sparus aurata*^86^ and *Rhabdosargus sarba*^58^, exhibit a surprisingly high GSI of 4.4% which, albeit lower than values of group spawning sparids (5.5%), is still higher than most gonochoristic species (3%). Therefore, while spawning in aggregations can explain the high GSI of many gonochoristic and protogynous species, mating system and spawning mode alone cannot explain the high GSI consistently found in protandrous sparids. This is not unique to the sparids. For example, the majority of damselfishes (family Pomacentridae) reproduce in pairs and, as predicted by SAM^14^, exhibit lower GSI values (max. GSI < 1%^87^); however, other protandrous damselfishes, such as the yellowtail clownfish *Amphiprion clarkii*, reproduce in pairs^88^ but exhibit unexpectedly high GSI (4.14%)^89^. This corroborates the suggestion that protandrous species can exhibit high GSI values, regardless of mating system.

Here we propose that it is precisely the nature of protandry what explains the high GSI in protandrous sparids with pair mating. Specifically, protandrous males (first sex) are always smaller than females. Thus, given that fecundity increases with size in females^90^, small protandrous males need to produce large amounts of sperm to effectively fertilize highly fecund females much larger than themselves, even when mating in pairs and under conditions of low levels of sperm competition. To do so successfully, they need to invest disproportionally in the gonads. For small-sized protandrous males, there might be a body size threshold below which the GSI has to increase to ensure enough sperm production to fertilize the eggs produced by the larger females. Consistently, evidence of sperm production adjustment in relation to the amount of eggs to be fertilized has been reported in a coral reef fish, *Thalassoma bifasciatum*^91^. Further, in many species with alternative male mating strategies, smaller “sneaker” males invest more in the gonads than larger territorial males^38^ due to the competitive environment as well as to compensate for their reduced size. Taken together, this evidence supports our suggestion that protandrous males have higher GSI than expected because they need to ensure the fertilization of eggs produced by much larger females than themselves.

The potential limitation in the fertilization capacity of small males is in agreement with the current debate questioning Bateman’s principles^92–94^. Briefly, Bateman principles state that, due to the smaller cost of producing sperm when compared to eggs: (i) male reproductive success (RS) increases with mate number whereas female RS does not; (ii) males have greater variance in RS than females, and (iii) the sex with the greater variance in RS undergoes stronger sexual selection^92,94^. Bateman’s principles have been used to test the strength of sexual selection between the sexes in gonochoristic^95^ and simultaneous hermaphroditic species, although whether they really apply to the latter is currently highly debated^93^. Surprisingly, although the presence of sexual selection in sequential hermaphrodites has been recognized with the assumption that it is stronger on the most abundant sex^96^, Bateman’s principles have not been formally tested. We argue that it is the combination of the existence of male-skewed sex ratios and male-male competition, on the one hand, and the problem for small males of producing enough sperm to fertilize large females, on the other hand, that together explain the high GSI in males of protandrous sparids, depending on the social/mating system. In fact, there is evidence that sperm depletion is a problem for many males across several taxa (reviewed in^94^). For example, in the simultaneous hermaphrodite polychaete *Ophryotrocha diadema*, small protandrous males can have difficulties fertilizing a full clutch of eggs^97^. Sperm depletion has been also documented in fish^98,99^. Moreover, in external spawners as most fishes are, mating rates (how many females can be fertilized) and ejaculation rates (how many sperm should be released in the water) should also be considered potential causes of higher GSI^100^. Thus, a higher than expected GSI may be related not only to sperm competition, which would relate to strong sexual selection in males in protandrous species spawning in groups, but also to a physiological compensatory mechanism that allows males to fertilize the many eggs that large females produce.

To test these ideas further, field studies are needed to corroborate whether the protandrous sparids with the highest GSI values are those that spawn in large groups or aggregations rather than in random matings or pairs. Furthermore, laboratory experiments aimed at determining the actual fertilization capacity of small-sized protandrous sparids, specifically fertilization rates with different amounts of sperm, will be key to determine whether there is indeed a size threshold below which the GSI needs to increase in order to ensure fertilization of the eggs released by the larger females. This evidence would advance substantially our understanding of the relationship between sexual systems and mating systems in this diverse family in particular and in teleosts in general.

To conclude, this study provides the most updated analysis of the distribution and incidence of different sexual systems in the family Sparidae, an ideal model taxon in which to study the evolution of sexual systems and SAM predictions. We have found that both protandry and protogyny can evolve from gonochorism and back, but evolutionary transitions between the two types of sequential hermaphroditism are unlikely if ever to occur. We show that the predictions of the SAM incorporating mating system, spawning mode and sperm competition, hold well for protogynous and gonochoristic species. In contrast, protandrous species do not conform to theoretical expectations. The high GSI values of some protandrous sparids suggest that males compete to fertilize the eggs of females while others mate in pairs in the absence of male-male competition but still invest greatly in gonad tissue. We suggest that this is due to a compensatory mechanism that is intrinsic to protandry: boosting male investment in the gonads to ensure successful fertilization of the considerable number of eggs released by highly fecund females that are much larger than protandrous males.

## Supplementary information

Suppl. Info.

## Data Availability

The datasets supporting this article have been uploaded as part of the supplementary material.
