# Supplementary material for: A phylogenetic comparative analysis on the evolution of sequential hermaphroditism in seabreams (Teleostei: Sparidae)
Source: Sci Rep. 2020 Feb 27;10:3606. doi: 10.1038/s41598-020-60376-w (PMC7046777; doi:10.1038/s41598-020-60376-w)
Supplement: Supplementary file 1 — Suppl. Info. [file 41598_2020_60376_MOESM1_ESM.docx]

Supplementary information

A phylogenetic comparative analysis on the evolution of sequential hermaphroditism in seabreams (Teleostei: Sparidae)

Susanna Pla, Chiara Benvenuto, Isabella Capellini and Francesc Piferrer

**Table S1.** List of the 68 sparid species used in this study, with information on their sexual system retrieved from FishBase and checked against the primary literature

| **Species** | **First description** | **Sexual system** | **Reference** |
| --- | --- | --- | --- |
| *Acanthopagrus australis* | Günther, 1859 | PA | Kailola *et al*., 1993 |
| *Acanthopagrus berda* | Forsskål, 1775 | PA | Tobin *et al*., 1997 |
| *Acanthopagrus bifasciatus* | Forsskål, 1775 | PA | Buxton & Garratt, 1990 |
| *Acanthopagrus butcheri* | Munro, 1949 | G | Kailola *et al*., 1993 |
| *Acanthopagrus latus* | Houttuyn, 1782 | PA | Buxton & Garratt, 1990 |
| *Acanthopagrus schlegelii* | Bleeker, 1854 | PA | Breder & Rosen, 1966 |
| *Archosargus rhomboidalis* | Linnaeus, 1758 | G | Chavance *et al*., 1986 |
| *Argyrops filamentosus* | Valenciennes, 1830 | G | Jayabalan *et al*., 2011 |
| *Argyrops spinifer* | Forsskål, 1775 | PG | El-Sayed & Abdel-Bary, 1993 |
| *Boops boops* | Linnaeus, 1758 | PG | Bauchot & Hureau, 1986 |
| *Boopsoidea inornata* | Castelnau, 1861 | G | Buxton & Garratt, 1990 |
| *Calamus arctifrons* | Goode & Bean, 1882 | G | Darcy, 1986 |
| *Calamus bajonado* | Bloch & Schneider, 1801 | PG | García-Cagide *et al*., 1994 |
| *Calamus leucosteus* | Jordan & Gilbert, 1885 | PG | Waltz *et al*., 1982 |
| *Calamus nodosus* | Randall & Caldwell, 1966 | PG | Horvath *et al*., 1990 |
| *Calamus penna* | Valenciennes, 1830 | G | Darcy *et al*., 1986 |
| *Calamus pennatula* | Guichenot, 1868 | PG | García-Cagide *et al*., 1994 |
| *Calamus proridens* | Jordan & Gilbert, 1884 | PG | García-Cagide *et al*., 1994 |
| *Centracanthus cirrus* | Rafinesque, 1810 | G | Tortonese, 1986 |
| *Cheimerius nufar* | Valenciennes, 1830 | G | Garratt, 1991 |
| *Chrysoblephus cristiceps* | Valenciennes, 1830 | PG | van der Elst & Adkin, 1991 |
| *Chrysoblephus gibbiceps* | Valenciennes, 1830 | G | Garratt, 1986 |
| *Chrysoblephus laticeps* | Valenciennes, 1830 | PG | van der Elst & Adkin, 1991 |
| *Chrysoblephus puniceus* | Gilchrist & Thompson, 1908 | PG | Garratt, 1986 |
| *Dentex canariensis* | Steindachner, 1881 | G | Bauchot & Hureau, 1986 |
| *Dentex dentex* | Linnaeus, 1758 | G | Bauchot & Hureau, 1986 |
| *Dentex gibbosus* | Rafinesque, 1810 | PG | Bauchot *et al*., 1981 |
| *Dentex macrophthalmus* | Bloch, 1791 | G | Bauchot & Hureau, 1986 |
| *Dentex maroccanus* | Valenciennes, 1830 | G | Bauchot & Hureau, 1986 |
| *Dentex tumifrons* | Temminck & Schlegel, 1843 | PG | Buxton & Garratt, 1990 |
| *Diplodus annularis* | Linnaeus, 1758 | PA | Salekhova, 1961 |
| *Diplodus argenteus* | Valenciennes, 1830 | PA | David *et al*., 2005 |
| *Diplodus bellottii* | Steindachner, 1882 | G | Bauchot & Hureau, 1986 |
| *Diplodus capensis* | Smith, 1844 | PA | David *et al*., 2005 |
| *Diplodus cervinus* | Lowe, 1838 | G | Bauchot & Hureau, 1986 |
| *Diplodus puntazzo* | Walbaum, 1792 | G | Garratt, 1986 |
| *Diplodus sargus cadenati* | de la Paz, Bauchot & Daget, 1974 | PA | Pajuelo & Lorenzo, 2004 |
| *Diplodus sargus kotschyi* | Steindachner, 1876 | PA | Abou-Seedo *et al*., 1990 |
| *Diplodus sargus sargus* | Linnaeus, 1758 | PA | Bauchot & Hureau, 1986 |
| *Diplodus vulgaris* | Geoffroy Saint-Hilaire, 1817 | G | Bauchot & Hureau, 1986 |
| *Lagodon rhomboides* | Linnaeus, 1766 | G | Cody & Bortone 1992 |
| *Lithognathus aureti* | Smith, 1962 | PA | Buxton & Garratt, 1990 |
| *Lithognathus mormyrus* | Linnaeus, 1758 | PA | Bauchot & Hureau, 1986 |
| *Oblada melanura* | Linnaeus, 1758 | G | Buxton & Garratt, 1990 |
| *Pachymetopon aeneum* | Gilchrist & Thompson, 1908 | PG | van der Elst & Adkin, 1991 |
| *Pachymetopon grande* | Günther, 1859 | G | Buxton & Garratt, 1990 |
| *Pagellus acarne* | Risso, 1827 | PA | Bauchot *et al*., 1981 |
| *Pagellus bellottii* | Steindachner, 1882 | PG | Bauchot *et al*., 1981 |
| *Pagellus bogaraveo* | Brünnich, 1768 | PA | de Mitcheson & Liu, 2008 |
| *Pagellus erythrinus* | Linnaeus, 1758 | PG | Buxton & Garratt, 1990 |
| *Pagrus auratus* | Forster, 1801 | G | Kailola *et al*., 1993 |
| *Pagrus aurigaa* | Valenciennes, 1843 | PG | Alekseev, 1982 |
| *Pagrus caeruleostictus* | Valenciennes, 1830 | PG | Buxton & Garratt, 1990 |
| *Pagrus major* | Temminck & Schlegel, 1843 | G | Buxton & Garratt, 1990 |
| *Pagrus pagrus* | Linnaeus, 1758 | PG | Pajuelo & Lorenzo, 1996 |
| *Petrus rupestris* | Valenciennes, 1830 | G | Buxton & Garratt, 1990 |
| *Polysteganus undulosus* | Regan, 1908 | G | Buxton & Garratt, 1990 |
| *Pterogymnus laniarius* | Valenciennes, 1830 | G | Hecht & Baird, 1977 |
| *Rhabdosargus globiceps* | Valenciennes, 1830 | PG | van der Elst & Adkin, 1991 |
| *Rhabdosargus sarba* | Forsskål, 1775 | PA | de Mitcheson & Liu, 2008 |
| *Sarpa salpa* | Linnaeus, 1758 | PA | Bauchot & Hureau, 1986 |
| *Sparidentex hasta* | Valenciennes, 1830 | PA | Lone *et al*., 2003 |
| *Sparodon durbanensis* | Castelnau, 1861 | G | Buxton & Garratt, 1990 |
| *Sparus aurata* | Linnaeus, 1758 | PA | Bauchot *et al*., 1981 |
| *Spicara maena* | Linnaeus, 1758 | PG | Tortonese, 1986 |
| *Spicara smaris* | Linnaeus, 1758 | PG | Tsangridis & Filippousis, 1992 |
| *Spondyliosoma cantharus* | Linnaeus, 1758 | PG | Bauchot & Hureau, 1986 |
| *Spondyliosoma emarginatum* | Valenciennes, 1830 | G | Smith & Smith, 1986 |

Abbreviations: G=Gonochorism; PA=Protandry; PG=Protogyny.

**Table S2.** List of sparid genera with known sexual system

| Genus | No. of species | Gonochorism (G) | Hermaphroditism | |
| --- | --- | --- | --- | --- |
|  |  |  | **Protandry (PA)** | **Protogyny (PG)** |
| *Acanthopagrus* | 20 | 1 | 5 | - |
| *Archosargus* | 3 | 1 | - | - |
| *Argyrops* | 4 | 1 | - | 1 |
| *Boops* | 2 | - | - | 1 |
| *Boopsoidea* | 1 | 1 | - | - |
| *Calamus* | 13 | 2 | - | 5 |
| *Centroacanthus* | 1 | 1 | - | - |
| *Cheimerius* | 2 | 1 | - | - |
| *Chrysoblephus* | 6 | 1 | - | 3 |
| *Dentex* | 13 | 4 | - | 2 |
| *Diplodus* | 15 | 4 | 6 | - |
| *Lagodon* | 1 | 1 | - | - |
| *Lithognathus* | 4 | - | 2 | - |
| *Oblada* | 1 | 1 | - | - |
| *Pachymetopon* | 3 | 1 | - | 1 |
| *Pagellus* | 6 | - | 2 | 2 |
| *Pagrus* | 6 | 2 | - | 3 |
| *Petrus* | 1 | 1 | - | - |
| *Polysteganus* | 8 | 1 | - | - |
| *Pterogymnus* | 1 | 1 | - | - |
| *Rhabdosagrus* | 6 | - | 1 | 1 |
| *Sarpa* | 1 | - | 1 | - |
| *Sparidentex* | 4 | - | 1 | - |
| *Sparodon* | 1 | 1 | - | - |
| *Sparus* | 1 | - | 1 | - |
| *Spicara* | 8 | - | - | 2 |
| *Spondyliosoma* | 2 | 1 | - | 1 |
| (10 other genera) | 14 |  |  |  |
| 37 | **148** | **27** | **19** | **22** |

**Table S3**. Male gonadosomatic index (GSI) values of the sparid species used in this study

| **Species** | **Sexual system** | **GSI** | **In Rabosky**  **tree 2018** | **In**  **Santini**  **tree** | **Reference** | **Spawning mode** | **Refs.** |
| --- | --- | --- | --- | --- | --- | --- | --- |
| *Acanthopagrus australis* | PA | 5.0 | Yes | Yes | ^1^ |  |  |
| *Acanthopagrus berda* | PA |  | Yes | Yes |  | GS | ^2,3^ |
| *Acanthopagrus bifasciatus* | PA |  | Yes | Yes |  |  |  |
| *Acanthopagrus butcheri* | G | 6.8 | Yes | No | ^4^ |  |  |
| *Acanthopagrus latus* | PA | 5.0 | Yes | Yes | ^5^ |  |  |
| *Acanthopagrus schlegelii* | PA | 3.4 | Yes | Yes | ^6^ |  |  |
| *Archosargus rhomboidalis* | G |  | Yes | Yes |  |  |  |
| *Argyrops filamentosus* | G | 1.3 | Yes | Yes | ^7^ |  |  |
| *Argyrops spinifer* | PG | 1.1 | Yes | Yes | ^8^ |  |  |
| *Boops boops* | PG | 4.0 | Yes | Yes | ^9^ |  |  |
| *Boopsoidea inornata* | G |  | No | Yes |  |  |  |
| *Calamus arctifrons* | G |  | No | No |  |  |  |
| *Calamus bajonado* | PG |  | No | No |  |  |  |
| *Calamus leucosteus* | PG |  | No | No |  |  |  |
| *Calamus nodosus* | PG | 0.1 | Yes | Yes | ^10^ |  |  |
| *Calamus penna* | G |  | Yes | Yes |  |  |  |
| *Calamus pennatula* | PG |  | No | No |  |  |  |
| *Calamus proridens* | PG |  | No | No |  |  |  |
| *Cheimerius nufar* | G | 1.3 | Yes | Yes | ^11^ | GS | ^2,12^ |
| *Centracanthus cirrus* | G |  | No | Yes |  |  |  |
| *Chrysoblephus cristiceps* | PG | 0.5 | Yes | Yes | ^12^ |  |  |
| *Chrysoblephus gibbiceps* | G |  | Yes | No |  |  |  |
| *Chrysoblephus laticeps* | PG | 1.2 | Yes | Yes | ^12^ | PS | ^12^ |
| *Chrysoblephus puniceus* | PG |  | Yes | Yes |  |  |  |
| *Dentex canariensis* | G |  | Yes | Yes |  |  |  |
| *Dentex dentex* | G | 4.0 | Yes | Yes | ^13^ |  |  |
| *Dentex gibbosus* | PG | 2.6 | Yes | Yes | ^14^ |  |  |
| *Dentex macrophthalmus* | G | 4.5 | Yes | Yes | ^15^ |  |  |
| *Dentex maroccanus* | G | 0.8 | Yes | Yes | ^16^ |  |  |
| *Dentex tumifrons* | PG | 1.8 | Yes | Yes | ^17^ |  |  |
| *Diplodus annularis* | PA | 5.0 | Yes | Yes | ^18^ | GS | ^19^ |
| *Diplodus argenteus* | PA | 6.0 | Yes | Yes | ^20^ |  |  |
| *Diplodus bellottii* | G | 4.4 | Yes | Yes | ^21^ |  |  |
| *Diplodus capensis* | PA | 5.5 | No | No | ^22^ | GS | ^22^ |
| *Diplodus cervinus* | G | 2.6 | Yes | Yes | ^23^ | GS | ^22^ |
| *Diplodus puntazzo* | G | 3.8 | Yes | Yes | ^24^ |  |  |
| *Diplodus sargus cadenati* | PA | 5.0 | Yes | No | ^25^ |  |  |
| *Diplodus sargus kotschyi* | PA | 4.1 | Yes | No | ^26^ |  |  |
| *Diplodus sargus sargus* | PA | 4.8 | Yes | Yes | ^18^ |  |  |
| *Diplodus vulgaris* | G | 3.0 | Yes | Yes | ^18^ |  |  |
| *Lagodon rhomboides* | G | 5.0 | Yes | Yes | ^27^ |  |  |
| *Lithognathus aureti* | PA |  | Yes | No |  |  |  |
| *Lithognathus mormyrus* | PA | 5.8 | Yes | Yes | ^28^ |  |  |
| *Oblada melanura* | G |  | Yes | Yes |  |  |  |
| *Pachymetopon aeneum* | PG | 1.9 | Yes | Yes | ^29^ |  |  |
| *Pachymetopon grande* | G |  | Yes | Yes |  |  |  |
| *Pagellus acarne* | PA | 3.5 | Yes | Yes | ^30^ |  |  |
| *Pagellus bellottii* | PG | 2.2 | Yes | Yes | ^31^ |  |  |
| *Pagellus bogaraveo* | PA | 2.0 | Yes | Yes | ^32^ |  |  |
| *Pagellus erythrinus* | PG | 2.0 | Yes | Yes | ^33^ |  |  |
| *Pagrus auratus* | G | 5.5 | Yes | Yes | ^34^ |  |  |
| *Pagrus auriga* | PG | 2.0 | Yes | Yes | ^35^ |  |  |
| *Pagrus caeruleostictus* | PG | 1.5 | Yes | Yes | ^36^ |  |  |
| *Pagrus major* | G | 7.5 | No | Yes | ^37^ |  |  |
| *Pagrus pagrus* | PG | 4.8 | Yes | Yes | ^38^ |  |  |
| *Petrus rupestris* | G | 1.5 | Yes | Yes | ^39^ |  |  |
| *Polysteganus undulosus* | G |  | Yes | No |  |  |  |
| *Pterogymnus laniarius* | G | 1.2 | Yes | Yes | ^40^ |  |  |
| *Rhabdosargus globiceps* | PG | 3.2 | Yes | Yes | ^41^ |  |  |
| *Rhabdosargus sarba* | PA | 4.0 | Yes | Yes | ^42^ | PS | ^43^ |
| *Sarpa salpa* | PA | 6.0 | Yes | Yes | ^44^ | GS | ^45^ |
| *Sparidentex hasta* | PA | 2.3 | Yes | Yes | ^46^ |  |  |
| *Sparodon durbanensis* | G | 5.0 | Yes | Yes | ^12^ |  |  |
| *Sparus aurata* | PA | 4.8 | Yes | Yes | ^47^ | PS | ^48^ |
| *Spicara maena* | PG | 1.8 | Yes | Yes | ^49^ |  |  |
| *Spicara smaris* | PG |  | Yes | Yes |  |  |  |
| *Spondyliosoma cantharus* | PG | 1.1 | Yes | Yes | ^50^ |  |  |
| *Spondyliosoma emarginatum* | G | 1.8 | Yes | No | ^51^ | PS | ^12^ |

Abbreviations: G = Gonochorism; PA = Protandry; PG = Protogyny; PS = Pair spawning; GS = Group spawning.

**Table S4.** Phylogenetic analysis of male life-history traits according to sexual systems (G, PA, PG) from the Santini *et al*. (2014) phylogenetic tree

| **Variables** | |  |  |  |  | **Model stats** | |
| --- | --- | --- | --- | --- | --- | --- | --- |
| **Dependent** | **Independent** | **Beta** | **T** | **P** | **df** | **λ** | **R^2^** |
| Weight (log_10_  transformed) | Sexual system - PA^1^ | 0.21 | 0.97 | 0.33 | 2,26 | 0.91 | 0.07 |
|  | Sexual system - PG^1^ | -0.16 | -0.88 | 0.38 | 2,26 |  |  |
|  | Sexual system - PG^2^ | -0.37 | -1.41 | 0.16 | 2,26 |  |  |
| Length (log_10_ transformed) | Sexual system - PA^1^ | -0.03 | -0.48 | 0.63 | 2,40 | 0.67 | 0.05 |
|  | Sexual system - PG^1^ | -0.10 | -1.57 | 0.12 | 2,40 |  |  |
|  | Sexual system - PG^2^ | -0.06 | -0.71 | 0.48 | 2,40 |  |  |
| Length at maturity  (log_10_ transformed) | Sexual system - PA^1^ | -0.12 | -1.54 | 0.13 | 2,23 | 0.00 | 0.10 |
|  | Sexual system - PG^1^ | -0.10 | -1.28 | 0.21 | 2,23 |  |  |
|  | Sexual system - PG^2^ | 0.02 | 0.29 | 0.76 | 2,23 |  |  |
| GSI (%) | Sexual system - PA^1^ | 0.99 | 1.71 | 0.09 | 2,41 | 0.00 | 0.31 |
|  | Sexual system - PG^1^ | -1.44 | -2.61 | **0.01** | 2,41 |  |  |
|  | Sexual system - PG^2^ | -2.44 | -4.26 | **0.00** | 2,41 |  |  |

Abbreviations: df = degrees of freedom; T, t-value; P = *p*-value; λ = phylogenetic signal; G = Gonochorism; PA = Protandry; PG = Protogyny. Significant differences are indicated in bold.

^1^ G as reference level.

^2^ PA as reference level.

|  | | Rabosky *et al.* (2018) | | | | | | Santini *et al.* (2014) | | | | | | |
| --- | --- | --- | --- | --- | --- | --- | --- | --- | --- | --- | --- | --- | --- | --- |
| **Variable statistics** | |  |  |  |  | **Model stats** | |  |  |  |  | **Model stats** | |  |
| **Dependent** | **Independent** | **Beta** | **T** | **P** | **df** | **λ** | ***R*^2^** | **Beta** | **T** | **P** | **df** | **λ** | ***R*^2^** |  |
| GSI (full ANCOVA model) | Length | 2.02 | -0.96 | 0.34 | 5,29 | 0.35 | 0.20 | 1.63 | 0.78 | 0.43 | 5,26 | 0.10 | 0.30 |  |
|  | PA | 6.18 | 0.91 | 0.36 | 5,29 |  |  | 8.02 | 1.09 | 0.28 | 5,26 |  |  |  |
|  | PG | -0.82 | -0.10 | 0.92 | 5,29 |  |  | -0.35 | -0.05 | 0.95 | 5,26 |  |  |  |
|  | Length*PA | -2.94 | -0.75 | 0.45 | 5,29 |  |  | -3.72 | -0.88 | 0.38 | 5,26 |  |  |  |
|  | Length*PG | -0.02 | -0.00 | 0.99 | 5,29 |  |  | -0.25 | -0.06 | 0.94 | 5,26 |  |  |  |
| GSI (without interactions) | Length | 1.35 | 0.80 | 0.42 | 3,31 | 0.27 | 0.19 | 0.38 | 0.21 | 0.82 | 3,21 | 0.00 | 0.31 |  |
|  | PA | 1.23 | 1.76 | 0.08 | 3,31 |  |  | 1.79 | 2.37 | **0.02** | 3,21 |  |  |  |
|  | PG | -0.90 | -1.40 | 0.16 | 3,31 |  |  | -0.23 | -0.32 | 0.74 | 3,21 |  |  |  |
| GSI (full ANCOVA model) | Weight | -0.63 | -0.72 | 0.47 | 5,19 | 0.00 | 0.47 | -1.35 | -1.30 | 0.20 | 5,19 | 0.71 | 0.45 |  |
|  | PA | -0.09 | -0.01 | 0.98 | 5,19 |  |  | -1.17 | -0.18 | 0.85 | 5,19 |  |  |  |
|  | PG | -10.26 | -1.57 | 0.13 | 5,19 |  |  | -9.46 | -1.55 | 0.13 | 5,19 |  |  |  |
|  | Weight *PA | 0.09 | 0.05 | 0.95 | 5,19 |  |  | 0.66 | 0.37 | 0.71 | 5,19 |  |  |  |
|  | Weight *PG | 2.16 | 1.23 | 0.23 | 5,19 |  |  | 1.84 | 1.14 | 0.26 | 5,19 |  |  |  |
| GSI (without interactions) | Weight | -0.18 | -0.27 | 0.78 | 3,21 | 0.00 | 0.43 | -0.71 | -0.90 | 0.37 | 3,21 | 0.00 | 0.42 |  |
|  | PA | 0.32 | 0.43 | 0.66 | 3,21 |  |  | 1.15 | 1.45 | 0.16 | 3,21 |  |  |  |
|  | PG | -2.27 | -3.16 | **0.00** | 3,21 |  |  | -2.55 | -3.25 | **0.00** | 3,21 |  |  |  |

**Table S5**. Results of the ANCOVA model of GSI against body length or weight and sexual systems (G, PA and PG) in Sparids using two different phylogenetic trees. Gonochorism is the reference level of the model

Abbreviations: df = degrees of freedom; T = t-value; λ = phylogenetic signal; P = *p*-value; *R*^2^  = Multiple R-squared; G = Gonochorism; PA = Protandry; PG = Protogyny.

**References in Table S3**

1 Pollock, B. Relations between migration, reproduction and nutrition in yellowfin bream *Acanthopagrus australis*. *Marine Ecology Progress Series*, 17-23 (1984).

2 Garratt, P. A. Comparative aspects of the reproductive biology of seabreams (Pisces: Sparidae). *Ph.D. Thesis, Rhodes University, Grahamstown,* **1**, 175 (1993).

3 Sheaves, M., Molony, B. & Tobin, A. Spawning migrations and local movements of a tropical sparid fish. *Marine Biology* **133**, 123-128, doi:<https://doi.org/10.1007/s002270050450> (1999).

4 Walker, S. & Neira, F. J. Aspects of the reproductive biology and early life history of black bream, *Acanthopagrus butcheri* (Sparidae), in a brackish lagoon system in southeastern Australia. *Journal of Ichthyology and Aquatic Biology* **4**, 135-142 (2001).

5 Abou‐Seedo, F., Dadzie, S. & Al‐Kanaan, K. Sexuality, sex change and maturation systems in the yellowfin seabream, *Acanthopagrus latus* (Teleostei: Sparidae)(Houttuyn, 1782). *Journal of Applied Ichthyology* **19**, 65-73, doi:<https://doi.org/10.1046/j.1439-0426.2003.00355.x> (2003).

6 Law, C. & Sadovy de Mitcheson, Y. Reproductive biology of black seabream Acanthopagrus schlegelii, threadfin porgy Evynnis cardinalis and red pargo Pagrus major in the northern South China Sea with consideration of fishery status and management needs. *Journal of Fish Biology* (2017).

7 Jayabalan, N., Al-Marzouqi, A. & Al-Nahdi, A. Reproductive biology of the soldierbream, *Argyrops filamentosus* (Valenciennes, 1830) from the Arabian Sea coast of Oman. *Indian Journal of Fisheries* **58**, 9-18 (2011).

8 Al Mamry, J., McCarthy, I., Richardson, C. & Ben Meriem, S. Biology of the kingsoldier bream (*Argyrops spinifer*, Forsskål 1775; Sparidae), from the Arabian Sea, Oman. *Journal of Applied Ichthyology* **25**, 559-564, doi:<https://doi.org/10.1111/j.1439-0426.2009.01260.x> (2009).

9 Dobroslavić, T., Mozara, R., Glamuzina, B. & Bartulović, V. Reproductive systems of bogue, *Boops boops* (Sparidae), in the southeastern Adriatic Sea. *Acta Adriatica* **58** (2017).

10 Horvath, M. L., Grimes, C. B. & Huntsman, G. R. Growth, mortality, reproduction and feeding of knobbed porgy, *Calamus nodosus*, along the southeastern United States coast. *Bulletin of marine science* **46**, 677-687 (1990).

11 Al‐Marzouqi, A. Biology of santer seabream *Cheimerius nufar* (Val. 1830) from the Arabian Sea off Oman. *Journal of Applied Ichthyology* **29**, 587-593 (2013).

12 Buxton, C. D. & Garratt, P. A. in *Alternative life-history styles of fishes* Vol. 10 (ed M.N. Bruton) Ch. Developments in environmental biology of fishes, 113-124 (Springer, 1990).

13 Grau, A. *et al.* Reproductive strategy of common dentex *Dentex dentex*: management implications. *Mediterranean Marine Science* **17**, 552-566, doi:<https://doi.org/10.12681/mms.1156> (2016).

14 Alves, A. Comparative analysis of the reproduction between two sparidae, *Pagrus pagrus* and *Dentex gibbosus*, caught of the Madeira Archipelago. (2010).

15 Soykan, O., Ilkyaz, A. T., Metin, G. & Kinacigil, H. T. Growth and reproduction of *Boops boops, Dentex macrophthalmus, Diplodus vulgaris,* and *Pagellus acarne* (Actinopterygii: Perciformes: Sparidae) from east-central Aegean Sea, Turkey. *Acta Ichthyologica et Piscatoria* **45**, 39-55 (2015).

16 Mohdeb, R. & Kara, M. H. Age, growth and reproduction of the Morocco dentex *Dentex maroccanus* of the eastern coast of Algeria. *Journal of the Marine Biological Association of the United Kingdom* **95**, 1261-1270, doi:<https://doi.org/10.1017/S0025315414001945> (2015).

17 Tominaga, O., Inoue, M., Kamata, M. & Seikai, T. Reproductive cycle of yellow sea bream *Dentex tumifrons* in Wakasa Bay, the Sea of Japan off central Honshu. *Fisheries Science* **71**, 1069-1077, doi:<https://doi.org/10.1111/j.1444-2906.2005.01065.x> (2005).

18 Mouine, N., Francour, P., Ktari, M. & Chakroun-Marzouk, N. Reproductive biology of four Diplodus species *Diplodus vulgaris, D. annularis, D. sargus sargus* and *D. puntazzo* (Sparidae) in the Gulf of Tunis (central Mediterranean). *Journal of the Marine Biological Association of the United Kingdom* **92**, 623-631, doi:<https://doi.org/10.1017/S0025315411000798> (2012).

19 Pajuelo, J. G. & Lorenzo, J. M. Edad y crecimiento del raspallón, *Diplodus annularis* (Pisces: Sparidae), en el archipiélago Canario (Atlántico centro-oriental). *Ciencias Marinas* **28** (2002).

20 David, G., Coutinho, R., Quagio-Grassiotto, I. & Verani, J. The reproductive biology of *Diplodus argenteus* (Sparidae) in the coastal upwelling system of Cabo Frio, Rio de Janeiro, Brazil. *African Journal of Marine Science* **27**, 439-447, doi:<https://doi.org/10.2989/18142320509504102> (2005).

21 Ndiaye, A. M. Study of sexual cycle and sexual inversion of *Diplodus bellottii* (Steindachner, 1882; Teleosteans: Sparidae) in Atlantic Ocean water on Senegalese coats. *IJRPB., July* **2**, 16-23 (2015).

22 Mann, B. & Buxton, C. The reproductive biology of *Diplodus sargus capensis* and *D. cervinus hottentotus* (Sparidae) off the south-east Cape coast, South Africa. *Cybium* **22**, 31-47 (1998).

23 Winkler, A., Santos, C. & Potts, W. Diagnosing the sexual system of *Diplodus cervinus hottentotus* (Pisces: Sparidae) from southern Angola. *African Journal of Marine Science* **36**, 505-512, doi:<https://doi.org/10.2989/1814232X.2014.969771> (2014).

24 Pajuelo, J., Lorenzo, J. & Domínguez‐Seoane, R. Gonadal development and spawning cycle in the digynic hermaphrodite sharpsnout seabream *Diplodus puntazzo* (Sparidae) off the Canary Islands, northwest of Africa. *Journal of Applied Ichthyology* **24**, 68-76, doi:<https://doi.org/10.1111/j.1439-0426.2007.01010.x> (2008).

25 Pajuelo, J. & Lorenzo, J. Basic characteristics of the population dynamic and state of exploitation of Moroccan white seabream *Diplodus sargus cadenati* (Sparidae) in the Canarian archipelago. *Journal of Applied Ichthyology* **20**, 15-21, doi:<https://doi.org/10.1046/j.0175-8659.2003.00540.x> (2004).

26 Abou-Seedo, F., Wright, J. M. & Clayton, D. Aspects of the biology of *Diplodus sargus kotschyi* (Sparidae) from Kuwait Bay. *Cybium. Paris* **14**, 217-223 (1990).

27 Nelson, G. A. Age, growth, mortality, and distribution of pinfish (*Lagodon rhomboides)* in Tampa Bay and adjacent Gulf of Mexico waters. *Fishery Bulletin* **100**, 582-592 (2002).

28 Eyman Faraj Abd, A., Sayed Mohamed, A., Mohammad El Sayed El Sayed El, M., Ramadan Attea Saleh, A. & Eman Salem Al, F. Reproductive Biology of the Striped Seabream *Lithognathus mormyrus* (Linnaeus, 1758) from Al Haneah Fishing Site, Mediterranean Sea, Eastern Libya. *Journal of Life Sciences* **10**, 171-181, doi:<https://doi.org/10.17265/1934-7391/2016.04.001> (2016).

29 Buxton, C. & Clarke, J. Age, growth and feeding of the blue hottentot *Pachymetopon aeneum* (Pisces: Sparidae) with notes on reproductive biology. *African Zoology* **21**, 33-38 (1986).

30 Arculeo, M., Brusle'‐Sicard, S., Potoschi, A. & Riggio, S. Investigations on gonadal maturation in *Pagellus acame* (Pisces, Sparidae) in the Strait of Messina (Sicily). *Italian Journal of Zoology* **67**, 333-337, doi:<https://doi.org/10.1080/11250000009356335> (2000).

31 Sadovy de Mitcheson, Y. & Liu, M. Functional hermaphroditism in teleosts. *Fish and Fisheries* **9**, 1-43, doi: <https://doi.org/10.1111/j.1467-2979.2007.00266.x> (2008).

32 Lechekhab, S., Lechekhab, H. & Djebar, B. Evolution of the hermaphrodite gonads during the sexual cycle of *Pagellus bogaraveo* (Sparidae) in Annaba Gulf, East coast of Algeria. *Cybium, International Journal of Ichthyology* **34**, 167-175 (2010).

33 Smida, M. A. B., Hadhri, N., Bolje, A. & Fehri-Bedoui, R. in *Annales: Series Historia Naturalis.* 31 (Scientific and Research Center of the Republic of Slovenia).

34 Scott, S. & Pankhurst, N. Interannual variation in the reproductive cycle of the New Zealand snapper *Pagrus auratus* (Bloch & Schneider)(Sparidae). *Journal of Fish Biology* **41**, 685-696, doi:<https://doi.org/10.1111/j.1095-8649.1992.tb02698.x> (1992).

35 Pajuelo, J. *et al.* Life history of the red‐banded seabream *Pagrus auriga* (Sparidae) from the coasts of the Canarian archipelago. *Journal of Applied Ichthyology* **22**, 430-436 (2006).

36 Ismail, R. F., Mourad, M. M. & Farrag, M. M. Gonadal development and hermaphroditism of bluespotted seabream, *Pagrus caeruleostictus* (Valenciennes, 1830) from the Mediterranean Sea, Egypt. *The Egyptian Journal of Aquatic Research* **44**, 163-171, doi:<https://doi.org/10.1016/j.ejar.2018.05.003> (2018).

37 Gen, K. *et al.* Unique expression of gonadotropin-I and-II subunit genes in male and female red seabream (*Pagrus major*) during sexual maturation. *Biology of reproduction* **63**, 308-319, doi:<https://doi.org/10.1095/biolreprod63.1.308> (2000).

38 Kokokiris, L., Bruslé, S., Kentouri, M. & Fostier, A. Sexual maturity and hermaphroditism of the red porgy *Pagrus pagrus* (Teleostei: Sparidae). *Marine Biology* **134**, 621-629, doi:<https://doi.org/10.1007/s002270050577> (1999).

39 Smale, M. Distribution and reproduction of the reef fish *Petrus rupestris* (Pisces: Sparidae) off the coast of South Africa. *African Zoology* **23**, 272-287 (1988).

40 Booth, A. J. & Buxton, C. D. The biology of the panga, *Pterogymnus laniarius* (Teleostei: Sparidae), on the Agulhas Bank, South Africa. *Environmental Biology of Fishes* **49**, 207-226, doi:<https://doi.org/10.1023/A:1007362700687> (1997).

41 Griffiths, M., Wilke, C., Penney, A. & Melo, Y. Life history of white stumpnose *Rhabdosargus globiceps* (Pisces: Sparidae) off South Africa. *African Journal of Marine Science* **24**, 281-300 (2002).

42 Hughes, J. M., Stewart, J., Kendall, B. W. & Gray, C. A. Growth and reproductive biology of tarwhine *Rhabdosargus sarba* (Sparidae) in eastern Australia. *Marine and Freshwater Research* **59**, 1111-1123, doi:<https://doi.org/10.1071/MF08102> (2009).

43 Leu, M.-Y. Natural spawning and larval rearing of silver bream, *Rhabdosargus sarba* (Forsskål), in captivity. *Aquaculture* **120**, 115-122, doi: <https://doi.org/10.1016/0044-8486(94)90227-5> (1994).

44 Paiva, R. B. *et al.* Age, growth and reproduction of the protandrous hermaphrodite fish, *Sarpa salpa*, from the Portuguese continental coast. *Journal of the Marine Biological Association of the United Kingdom*, 1-13, doi:<https://doi.org/10.1017/S0025315416001405> (2016).

45 Van der Walt, B. & Mann, B. Aspects of the reproductive biology of *Sarpa salpa* (Pisces: Sparidae) off the east coast of South Africa. *African Zoology* **33**, 241-248 (1998).

46 Lone, K., Ablani, S. & Al‐Yaqout, A. Steroid hormone profiles and correlative gonadal histological changes during natural sex reversal of sobaity kept in tanks and sea‐cages. *Journal of fish biology* **58**, 305-324, doi:<https://doi.org/10.1111/j.1095-8649.2001.tb02255.x> (2001).

47 Hadj-Taieb, A., Ghorbel, M., Hadj-Hamida, N. B. & Jarboui, O. Proporción de sexos, reproducción y crecimiento de la dorada, *Sparus aurata* (Pisces: Sparidae), en el golfo de Gabes, Túnez. *Ciencias marinas* **39**, 101-112, doi: <https://doi.org/10.7773/cm.v39i1.2146> (2013).

48 Ibarra-Zatarain, Z. & Duncan, N. Mating behaviour and gamete release in gilthead seabream (*Sparus aurata*, Linnaeus 1758) held in captivity. *Spanish Journal of Agricultural Research* **13**, e04-011, doi:<https://doi.org/10.5424/sjar/2015131-6750> (2015).

49 Soykan, O., İLKYAZ, A. T., METİN, G. & Kinacigil, H. T. Growth and reproduction of blotched picarel (*Spicara maena* Linnaeus, 1758) in the central Aegean Sea, Turkey. *Turkish Journal of Zoology* **34**, 453-459 (2010).

50 Pajuelo, J. G. & Lorenzo, J. M. Life history of black seabream, *Spondyliosoma cantharus*, off the Canary Islands, Central-east Atlantic. *Environmental Biology of fishes* **54**, 325-336, doi:<https://doi.org/10.1023/A:1007515301745> (1999).

51 Fairhurst, L., Attwood, C., Durholtz, M. & Moloney, C. Life history of the steentjie Spondyliosoma emarginatum (Cuvier 1830) in Langebaan Lagoon, South Africa. *African Journal of Marine Science* **29**, 79-92 (2007).

52 Rabosky, D. L. *et al.* Rates of speciation and morphological evolution are correlated across the largest vertebrate radiation. *Nature communications* **4**, doi: <https://doi.org/10.1038/ncomms2958> (2013).

53 Chiba, S. N., Iwatsuki, Y., Yoshino, T. & Hanzawa, N. Comprehensive phylogeny of the family Sparidae (Perciformes: Teleostei) inferred from mitochondrial gene analyses. *Genes & Genetic Systems* **84**, 153-170, doi: <https://doi.org/10.1266/ggs.84.153> (2009).
